# Evaluation of ebselen in resolving a methicillin-resistant *Staphylococcus aureus* infection of pressure ulcers in obese and diabetic mice

**DOI:** 10.1371/journal.pone.0247508

**Published:** 2021-02-22

**Authors:** Haroon Mohammad, Nader S. Abutaleb, Alexandra M. Dieterly, L. Tiffany Lyle, Mohamed N. Seleem

**Affiliations:** 1 Department of Comparative Pathobiology, College of Veterinary Medicine, Purdue University, West Lafayette, IN, United States of America; 2 Department of Biomedical Sciences and Pathobiology, Virginia-Maryland College of Veterinary Medicine, Virginia Polytechnic Institute and State University, Blacksburg, VA, United States of America; 3 Center for Comparative Translational Research, Purdue University, West Lafayette, IN, United States of America; Universidade Nova de Lisboa, PORTUGAL

## Abstract

Pressure ulcers (PUs) are a source of morbidity in individuals with restricted mobility including individuals that are obese or diabetic. Infection of PUs with pathogens, including methicillin-resistant *Staphylococcus aureus* (MRSA), impairs ulcers from healing. The present study evaluated ebselen as a topical antibacterial to treat MRSA-infected PUs. Against two different *S*. *aureus* strains, including MRSA USA300, resistance to ebselen did not emerge after 14 consecutive passages. Resistance to mupirocin emerged after only five passages. Additionally, ebselen was found to exert a modest postantibiotic effect of five hours against two MRSA strains. Ebselen was subsequently evaluated in MRSA-infected PUs in two models using obese and diabetic mice. In obese mice, topical ebselen (89.2% reduction) and oral linezolid (84.5% reduction) similarly reduced the burden of MRSA in infected PUs. However, in diabetic mice, topical ebselen (45.8% reduction in MRSA burden) was less effective. Histopathological evaluation of ulcers in diabetic mice determined that ebselen treatment resulted in fewer bacterial colonies deep within the dermis and that the treatment exhibited evidence of epithelial regeneration. Topical mupirocin was superior to ebselen in reducing MRSA burden in infected PUs both in obese (98.7% reduction) and diabetic (99.3% reduction) mice. Ebselen’s antibacterial activity was negatively impacted as the bacterial inoculum was increased from 10^5^ CFU/mL to 10^7^ CFU/mL. These results suggest that a higher dose of ebselen, or a longer course of treatment, may be needed to achieve a similar effect as mupirocin in topically treating MRSA-infected pressure ulcers.

## Introduction

Each year, over 2.5 million individuals develop pressure ulcers (PUs) in the U.S. alone resulting in total treatment costs estimated to range from $9 to $11 billion USD [1]. Ranging from mild to severe, PUs often develop in individuals with limited mobility including those who are obese, hospitalized, or residents in long-term care facilities. It is estimated that up to 12.1% of adult patients in hospital intensive care units (ICU) in the U.S. develop a PU annually; hospital-acquired PUs alone can result in total treatment costs that exceed $3 billion USD [2, 3]. As nearly 40% (>93 million) of the adult population in the U.S. is obese, and over 25% of patients admitted to the ICU are obese, there is a high probability for PUs to form in this patient population [4, 5]. Moreover, obesity is often associated with other co-morbidities, including type 2 diabetes. Individuals that are obese and/or diabetic are at greater risk for developing PUs, particularly in lower extremities, due to pathophysiological changes that occur that include vascular insufficiency, peripheral neuropathy, and breakdown of skin tissue [6, 7]. As a result, it is estimated that up to 34% of people with diabetes will develop a foot ulcer at least once in their lifetime [8].

Bacterial infections of PUs are a leading cause of hospital admissions in individuals that are obese and diabetic [6]. Infections can permeate to underlying tissues and bones resulting in osteomyelitis, sepsis, and can lead to amputation of infected limbs. It is estimated in the U.S., 15% of patients that experience a diabetic foot ulcer will undergo amputation of the affected limb, often due to a bacterial infection that interferes with granulation tissue formation and contributes to impaired wound healing [9–11]. *Staphylococcus aureus* and aerobic Gram-positive cocci are the leading sources of infection of PUs, particularly in the U.S. and in European nations [9, 12, 13]. *S*. *aureus* secretes toxins, including Panton–Valentine leukocidin (PVL), that result in tissue necrosis and formation of cutaneous abscesses [14]. Though systemic antibiotics such as clindamycin, linezolid, and vancomycin are recommended for the treatment of *S*. *aureus*-infected pressure ulcers, the emergence of strains of methicillin-resistant *S*. *aureus* (MRSA) has been linked to resistance to these agents, which has resulted in treatment failure [7, 15]. Treatment failure has also been linked to the ability of *S*. *aureus* to form robust biofilms in PUs, which many antibiotics are unable to penetrate to eradicate infection, resulting in impaired wound healing and chronic infection [9, 15]. Consequently, finding new agents capable of treating infected PUs are needed.

Ebselen, 2-phenyl-1,2-benzoisosenelazol-3(2H)-one, is an organoselenium compound first reported as a potent antioxidant in 1984 [16]. Originally called PZ-51, ebselen has been investigated in clinical trials for the treatment of stroke, oxidative stress in patients with diabetes, and hearing loss [17–20]. More recently, researchers have investigated the potential to reposition ebselen as an antibacterial agent, particularly against Gram-positive pathogens [21–26]. Ebselen was found to inhibit growth of *S*. *aureus*, including MRSA strains, *in vitro* (minimum inhibitory concentration (MIC) of 0.25 μg/mL against 90% of strains tested), successfully disrupted preformed *S*. *aureus* biofilms, and inhibited expression of PVL [25, 26]. In two independent rodent studies, ebselen significantly reduced the burden of MRSA when applied topically to acute uncomplicated skin wounds [23, 26]. In both rodent studies, treatment with topical ebselen resulted in a notable decrease in expression of proinflammatory cytokines including tumor necrosis factor-α (TNF-α), interleukin-6 (IL-6), and interleukin-1 beta (IL-1β) [23, 26]. Furthermore, treatment with topical ebselen resulted in improved wound healing relative to the controls [23]. To date, no studies have investigated the ability of topical ebselen to treat MRSA-infected pressure ulcers or other chronic wounds. We hypothesized that the potent antibacterial and antibiofilm activity against MRSA combined with its anti-inflammatory activity would make ebselen a promising novel topical agent to treat pressure ulcers infected with MRSA. Therefore, the aim of this study was to investigate ebselen’s ability to reduce the bacterial burden in MRSA-infected pressure ulcers formed in obese and diabetic mice.

## Results

### *S*. *aureus* did not develop resistance to ebselen after multiple exposures

**Fig 1:**
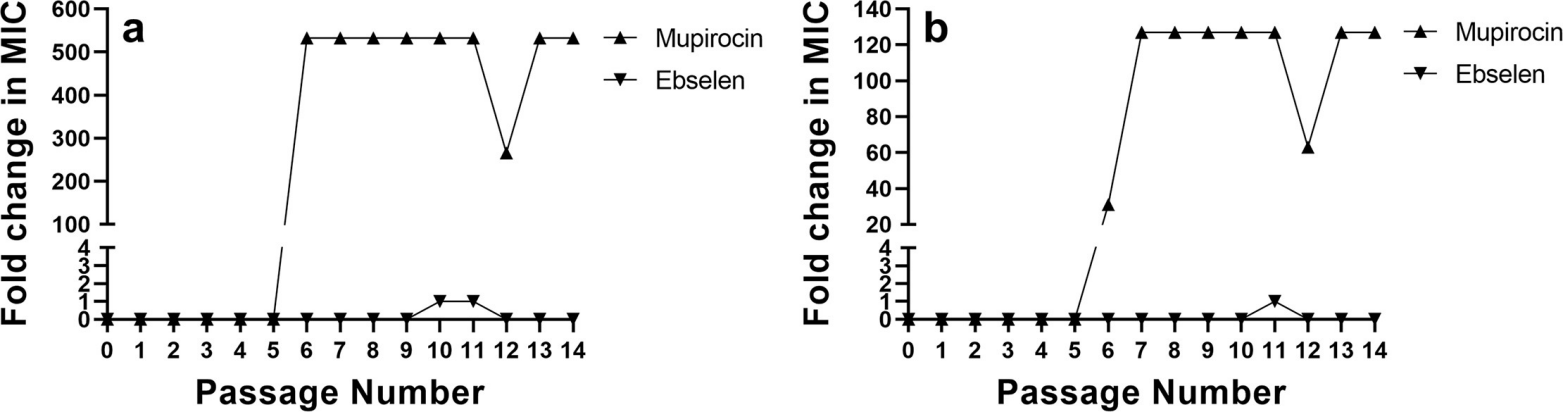
Multistep resistance selection for ebselen and mupirocin against two strains of *S*. *aureus*. Ebselen and mupirocin were passaged against (a) *S*. *aureus* ATCC 6538 and (b) methicillin-resistant *S*. *aureus* (MRSA USA300) for 14 days. The broth microdilution assay was used to determine the minimum inhibitory concentration (MIC) of ebselen and mupirocin after each passage. Data are presented as fold change in MIC relative to the initial MIC determined at passage 0.

A concern with the widespread use of antibiotics is the formation of resistance by certain bacterial species, including *S*. *aureus*, to these vital therapeutics. Resistance to topical antibiotics including mupirocin and fusidic acid has been observed, particularly in situations where use is widespread and access is not restricted, contributing to treatment failure [27]. To investigate the possibility of *S*. *aureus* developing resistance to ebselen after multiple exposures, a multi-step resistance selection assay was conducted against two strains of *S*. *aureus*. Resistance to ebselen was not observed against either *S*. *aureus* ATCC 6538 or MRSA USA300, even after 14 consecutive passages (Fig 1). A one-fold increase in the MIC of ebselen was observed after passage 10 (though the MIC returned back to the value observed in the initial passage after passage 11) against *S*. *aureus* ATCC 6538. Similarly, only a one-fold increase in the MIC value for ebselen was observed after passage 11 against MRSA USA300. In contrast, resistance to mupirocin emerged quickly. Against *S*. *aureus* ATCC 6538, mupirocin’s MIC increased 532-fold after the fifth passage and increased 127-fold after the sixth passage against MRSA USA300. The rapid development of resistance to mupirocin is in agreement with previous reports [28, 29].

### Ebselen exhibits a moderate postantibiotic effect against MRSA

The *in vitro* postantibiotic effect (PAE) of ebselen was determined against three strains of *S*. *aureus* exposed to the compound (at 5 × MIC) for one hour. Against *S*. *aureus* ATCC 6538, ebselen’s PAE exceeded nine hours (Table 1). When evaluated against MRSA USA300 and MRSA USA400, ebselen’s PAE was found to be five hours. Ebselen’s PAE against MRSA was slightly longer than the PAE observed for mupirocin (four hours), as we determined in a previous study [30]. The similar PAE observed for ebselen and mupirocin could potentially be due to the fact that both agents exert their effect, in part, by inhibiting bacterial protein synthesis albeit through binding to different molecular targets [24–26].

**Table 1:** Postantibiotic effect of ebselen (tested at 5 × MIC) against three different strains of *Staphylococcus aureus*.

Strain Designation	Postantibiotic effect (hours)
*S*. *aureus* ATCC 6538	> 9
MRSA USA300 (NRS384)	5
MRSA USA400 (NRS123)	5

### Evaluation of ebselen’s ability to reduce the burden of MRSA in infected pressure ulcers in mice

**Fig 2:**
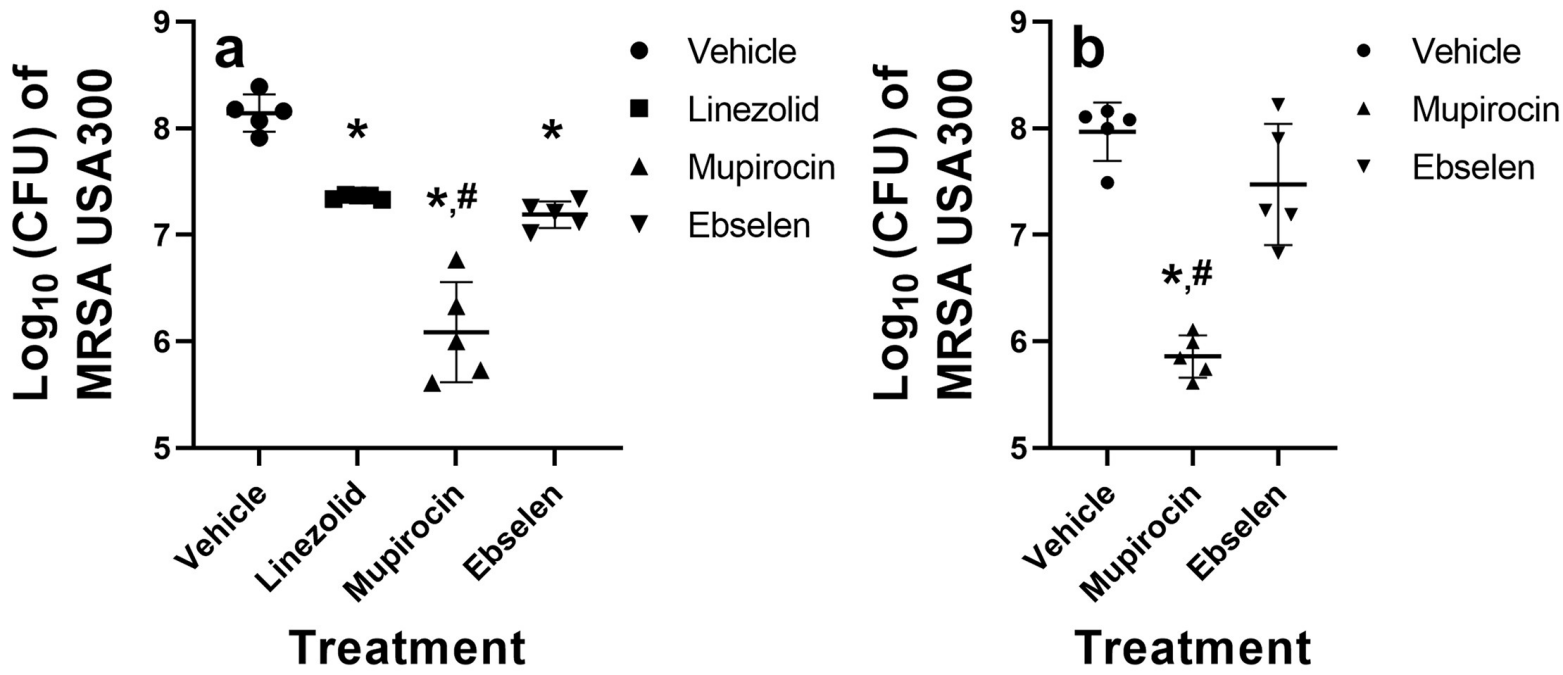
Evaluation of ebselen or mupirocin treatment of infected pressure ulcers in obese and diabetic TALLYHO/JngJ mice. The skin of female (obese) and male (diabetic) TALLYHO/JngJ mice (n = 5) were exposed to magnets to form pressure ulcers that were subsequently infected with MRSA USA300. Approximately 48 hours later, PUs were treated with oral linezolid (10 mg/kg, only for obese mice), topical mupirocin (2%), topical ebselen (2%) or vehicle alone (petroleum jelly) twice daily for either five (obese mice) or four (diabetic mice) days. Mice were humanely euthanized 12 hours after the final treatment dose and the infected PUs were aseptically removed to determine bacterial burden. (a) Reduction in burden of MRSA USA300 in pressure ulcers after treatment with the vehicle alone, linezolid, mupirocin, or ebselen in female obese TALLYHO/JngJ mice. (b) Reduction in burden of MRSA USA300 in pressure ulcers after treatment with the vehicle alone, mupirocin, or ebselen in male diabetic TALLYHO/JngJ mice. Data are presented as log_10_ (total MRSA CFU per wound) for each mouse and were evaluated using a one-way ANOVA with post-hoc Dunnet’s test for multiple comparisons. An asterisk (*) indicates statistical difference for test agents relative to petroleum jelly (negative control, *P* < 0.05) while a pound sign (#) indicates statistical difference between mice treated with mupirocin compared to ebselen (*P* < 0.05).

After confirming that MRSA is unlikely to develop resistance to ebselen after multiple exposures and determining the PAE of ebselen *in vitro*, we investigated the ability of ebselen to reduce the burden of MRSA in infected PUs both in obese and diabetic mice. First, PUs were developed and infected with a high inoculum of MRSA USA300 in obese female TALLYHO/JngJ mice. After five days of treatment with linezolid, mupirocin, or ebselen, mice were euthanized and the pressure ulcers were harvested to determine MRSA burden. Treatment of the infected PUs with topical mupirocin resulted in the largest decrease in bacterial burden (2.05-log_10_ reduction [98.7% reduction] relative to mice treated with the vehicle alone) (Fig 2A). Ebselen (0.95-log_10_ reduction [89.2% reduction]) reduced MRSA burden in infected PUs similar to treatment with oral linezolid (0.78-log_10_ reduction [84.5% reduction]) (*P* = 0.6178). Mupirocin (*P* < 0.0001), linezolid (*P* = 0.0006), and ebselen (*P* < 0.0001) all produced a statistically significant reduction in MRSA burden compared to the vehicle alone. Additionally, mupirocin was found to be superior to topical ebselen (*P* < 0.0001) in reducing the burden of MRSA in infected PUs of obese mice.

We next evaluated the ability of ebselen and mupirocin to reduce the burden of MRSA in infected PUs in male diabetic TALLYHO/JngJ mice (Fig 2B). Linezolid was excluded in this experiment as mupirocin exhibited more potent activity against MRSA-infected PUs in obese mice. Ebselen reduced the burden of MRSA by 0.50-log_10_ (45.8% reduction), which was not statistically significant relative to treatment with the vehicle alone (*P* = 0.0633). In contrast, mupirocin exhibited potent antibacterial activity and reduced the burden of MRSA by 2.11-log_10_ (99.3% reduction). Mupirocin was found to be superior to ebselen in reducing the burden of MRSA in infected PUs of diabetic mice (*P* < 0.0001).

### Histopathology evaluation of infected PUs treated with ebselen and mupirocin in diabetic mice

**Fig 3:**
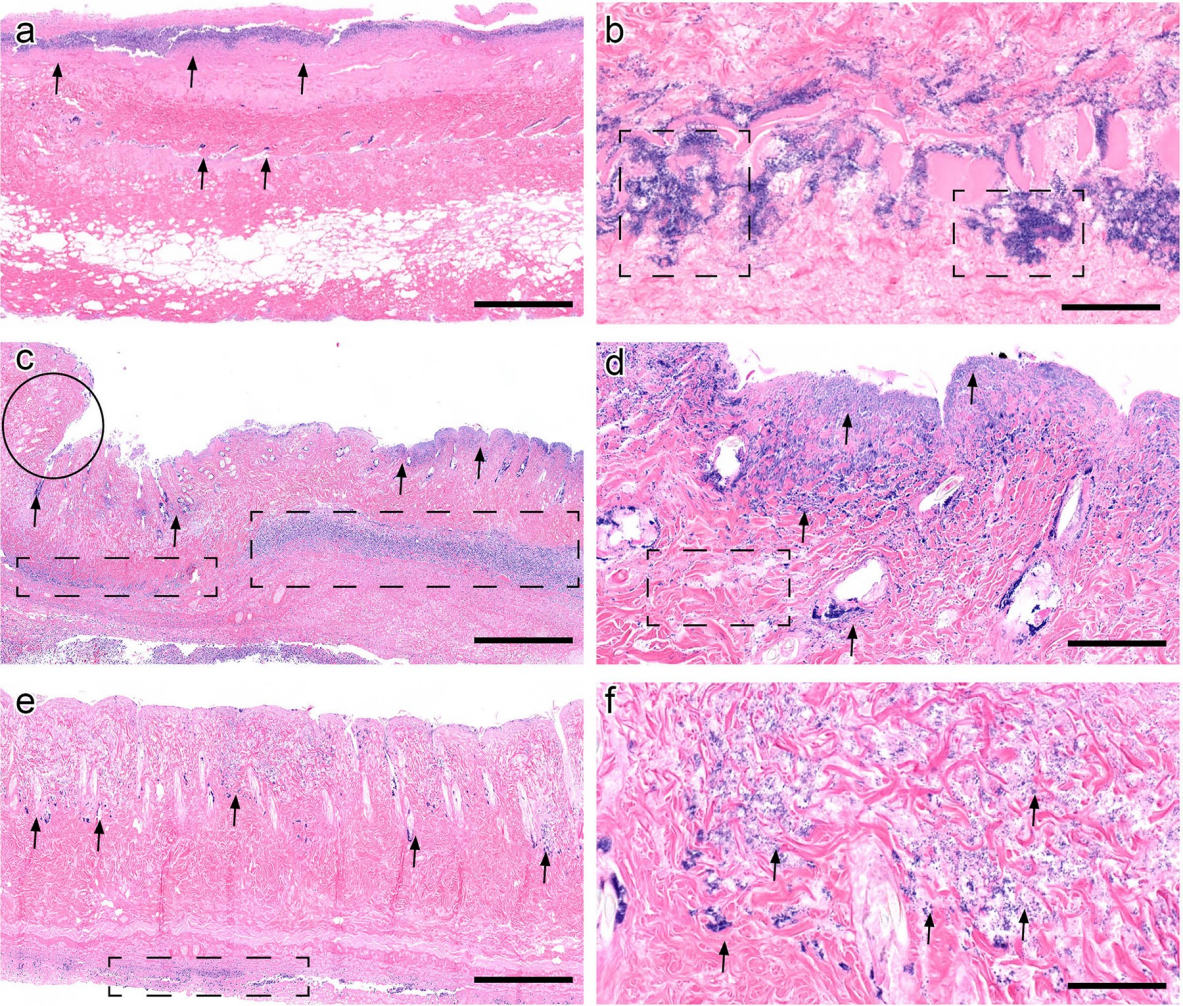
Histological evaluation of infected pressure ulcers in diabetic TALLYHO/JngJ mice. (a) Negative controls (vehicle alone) were characterized by dense thick carpets of superficial bacterial colonies (arrows) and full thickness necrosis, extending into the deep subcutis (image acquired at 2× magnification). Scale bar represents 1 mm. (b) On closer view, bacteria extend deep into the dermal tissue and are innumerable, forming thick aggregates (dashed boxes, image acquired at 20× magnification). Scale bar represents 100 μm. (c) Mupirocin-treated diabetic animals had a diminished bacterial burden, confined to the deep dermis and subcutis (arrows) surrounded by wound contraction (circle), indicating early wound healing, absent in negative controls. Inflammation extends deep into the subcutaneous tissue (dashed boxes) in contrast to untreated controls (image acquired at 2× magnification). Scale bar represents 1 mm. (d) On closer view, bacteria are less numerous and form small clusters compared to negative controls and collagen fibers are visible (dashed box). Scale bar represents 100 μm. (e) Ebselen-treated animals had a pronounced decrease in bacterial burden compared to the negative control, with few scattered aggregates in the deep dermis (arrows) and normal collagen fiber architecture in between. Inflammation was sparse compared to mupirocin treatment and present only at the deep tissue margin, indicating tissue clearing and early wound healing (dashed boxes) (image acquired at 2× magnification). Scale bar represents 1 mm. (f) Bacteria are sparse and markedly decreased compared to the negative control, with few colonies in between collagen fibers and occasionally forming clusters (arrows) (image acquired at 20× magnification).

In order to investigate if treatment with either ebselen or mupirocin impacted wound healing of infected PUs in diabetic mice, the affected tissues were analyzed with histopathology using hematoxylin and eosin staining. Negative controls were characterized by mats of innumerable superficial bacterial colonies (arrows) and full thickness necrosis, extending into the deep subcutaneous tissue and to the border of the image (Fig 3A). Bacterial colonies extended deep into the dermal tissue and were innumerable, forming thick clumps (Fig 3B, dashed boxes). Diabetic animals treated with mupirocin had fewer bacterial colonies confined to the deep dermis and subcutis (Fig 3C, arrows), bordered by contraction and granulation tissue formation (Fig 3C, circle), indicating early wound healing, absent in negative controls. Necrosis was less pronounced, with some collagen visible in the dermis and cellular detail present, again demonstrating early wound resolution. Inflammation extended deep into the subcutaneous tissue (Fig 3C, dashed boxes). On closer view, bacteria were much fewer and formed small clusters compared to untreated controls (Fig 3D, arrows). Additionally, collagen fibers were visible (Fig 3D, dashed box). Ebselen-treated animals had a markedly diminished bacterial burden compared to the negative control, with few scattered aggregates in the deep dermis (Fig 3E, arrows) and normal collagen fiber architecture. Inflammation was similarly markedly decreased and present only at the deep tissue margin, indicating tissue clearing and signs of wound healing (Fig 3E, dashed boxes). On higher view, bacteria were sparse and markedly decreased compared to the negative control, with few colonies in between collagen fibers and occasionally forming clusters (Fig 3F, arrows). Scale bars represent 1 mm in 2× view images (Fig 3A, 3C and 3E) and 100 μm in 20× view images, respectively (Fig 3B, 3D and 3F).

### Impact of pH and inoculum size on the antibacterial activity of ebselen

Typically, the surface of healthy intact skin tends to be acidic [31]. However, in acute and chronic wounds, the pH of the wound bed can become either acidic or basic [31]. To examine, if a change in pH may have negatively impacted the antibacterial activity of ebselen in the mice study, the MIC was determined against three strains of *S*. *aureus* in media under standard (neutral pH, bacterial inoculum equivalent to ~10^5^ CFU/mL), acidic, and basic conditions. As presented in Table 2, the MIC of ebselen (0.25 μg/mL) against *S*. *aureus* was not affected by a change in pH to acidic (pH 6.0) or basic (pH 8.0) conditions. The MIC of ebselen increased slightly (one- to two-fold) when the media was adjusted to pH 9.0. In contrast, the MIC values for mupirocin improved eight- to 16-fold under acidic conditions compared to standard conditions, in agreement with previous reports [32, 33]. However, at higher pH values (pH 8.0 and 9.0), the MIC values of mupirocin increased by one- to eight-fold.

**Table 2:** Minimum inhibitory concentration (MIC in μg/mL) of ebselen and mupirocin determined against three strains of *Staphylococcus aureus* in the presence of different pH and high bacterial inoculum.

	EBSELEN							MUPIROCIN						
Bacterial Strain	Standard CAMHB^1^	pH 6.0	pH 8.0	pH 9.0	10^7^ CFU/mL, pH 7.4	10^7^ CFU/mL, pH 8.0	10^7^ CFU/mL, pH 9.0	Standard CAMHB^1^	pH 6.0	pH 8.0	pH 9.0	10^7^ CFU/mL, pH 7.4	10^7^ CFU/mL, pH 8.0	10^7^ CFU/mL, pH 9.0
*S*. *aureus* ATCC 6538	0.25	0.25	0.25	1	16	8	8	0.25	≤ 0.03	1	0.50	0.50	0.50	1
MRSA USA300 (NRS384)	0.25	0.25	0.25	0.50	16	8	8	0.50	≤ 0.03	2	1	1	1	4
MRSA USA400 (NRS123)	0.25	0.25	0.25	0.50	8	8	8	0.25	≤ 0.03	2	1	1	2	8

^1^ Standard cation-adjusted Mueller-Hinton broth (pH 7.4) and inoculum size (10^5^ CFU/mL)

We next assessed the impact of inoculum size on the antibacterial activity of ebselen. Standard microdilution broth assays for *S*. *aureus* use an inoculum size ~10^5^ CFU/mL. However, in our mice studies, a much higher inoculum was used to infect the pressure ulcers. Thus, the lack of efficacy observed for ebselen we postulated may be in part due to the higher inoculum size used. To investigate this point, the MIC of ebselen was determined against three strains of *S*. *aureus* grown under standard conditions and high inoculum (10^7^ CFU/mL) conditions. Interestingly, there was a noticeable loss in activity observed for ebselen against *S*. *aureus* ATCC 6538 (64-fold increase), MRSA USA300 (64-fold increase), and MRSA USA400 (32-fold increase) (Table 2). In contrast, the MIC of mupirocin only increased two- (against *S*. *aureus* ATCC 6538 and MRSA USA300) to four-fold (against MRSA USA400).

We also investigated the dual impact of increased bacterial inoculum size and basic pH values on the antibacterial activity of ebselen (Table 2). The MIC of ebselen was determined against three *S*. *aureus* strains in media adjusted to pH 8.0 or pH 9.0 and in the presence of high inoculum (10^7^ CFU/mL). There was a noticeable increase in the MIC values for ebselen against the three strains tested (32-fold increase), at both pH 8.0 and pH 9.0, when the bacterial inoculum was increased to 10^7^ CFU/mL. Interestingly, these results were comparable to the MIC of ebselen determined at neutral pH and high bacterial inoculum. This indicates that the loss in ebselen’s antibacterial activity is primarily due to the effect of increased inoculum size of *S*. *aureus*. Conversely, the MIC of mupirocin increased in response to both an increase in the bacterial inoculum size and pH. At neutral pH, in the presence of high bacterial inoculum, the MIC values for mupirocin ranged from 0.50–1 μg/mL. In the presence of high bacterial inoculum, the MIC values for mupirocin increased by two-fold (against *S*. *aureus* ATCC 6538) at pH 9.0, increased by four-fold (against MRSA USA300) at pH 9.0, and increased two- and eight-fold (against MRSA USA400) at pH 8.0 and pH 9.0, relative to the MIC values determined for mupirocin at neutral pH.

## Discussion

Pressure ulcers (PUs) are a significant source of morbidity and treatment costs to individuals with limited mobility particularly those who are hospitalized, obese, or diabetic. Bacterial infection of PUs impairs wound healing and can lead to a chronic infection that is unresponsive to treatment. The secretion of toxins by pathogens such as *S*. *aureus* can exacerbate damage to ulcerated lesions while the formation of biofilms can protect encased pathogens from the effect of antibiotics and the host immune response [9, 14, 15]. Guidelines provided by the Infectious Diseases Society of America recommends the use of oral or parenteral antibiotics, such as linezolid or vancomycin, for treatment of uncomplicated MRSA skin and soft tissue infections (SSTIs, including ulcers) [34]. However, topical antibiotics, such as 2% mupirocin ointment, are permitted in the case of mild-to-moderate SSTIs [9, 34]. Topical antibiotics afford certain purported advantages over systemic antibiotics, particularly to treat localized mild-to-moderate skin infections, including increased concentration of drug at the site of infection and avoiding systemic toxicity and gastrointestinal dysbiosis observed with oral and parenteral antibiotics [27]. Amongst the most widely prescribed antibiotics for skin ailments, there were 4.7 million prescriptions for topical antibiotics submitted by primary care physicians in the United Kingdom in 2015 alone [27]. Mupirocin is a key topical antibiotic used to treat MRSA skin and soft tissue infections; however, widespread use of mupirocin has been associated with the emergence of bacterial strains that exhibit high-level resistance to mupirocin (MIC ≥ 512 μg/mL) [35–38]. Finding alternative topical antibiotics capable of rapidly reducing bacterial burden would be ideal for treatment of mild-to-moderate infected pressure ulcers. With this point in mind, the aim of this study was to investigate the potential for ebselen to be repurposed as a topical antibiotic to treat MRSA-infected pressure ulcers.

In 1989, the first report of ebselen possessing antibacterial activity against MRSA was published [39]. However, more than a decade elapsed before another research group pursued additional work with ebselen as an antibacterial agent while other groups have attempted to generate more potent analogues with limited success [40–42]. Ebselen exerts its antibacterial activity by inhibiting bacterial protein synthesis and inhibiting thioredoxin reductase thus making bacteria more susceptible to oxidative stress [23, 24, 26]. In addition to its potent antibacterial activity, ebselen exhibits potent antibiofilm activity against *S*. *aureus* and is capable of inhibiting production of toxic shock syndrome toxin-1 and PVL [26]. Furthermore, ebselen has been shown to exhibit potent anti-inflammatory activity in two rodent models of MRSA skin infection reducing the expression of key cytokines (TNF-α, IL-1β, and IL-6) that impair wound healing [23, 26]. Bi *et al*. demonstrated that selenium inhibited both the NF-κB and MAPK signaling pathways in *S*. *aureus*-infected macrophages, which resulted in decreased expression of pro-inflammatory cytokines including TNF-α, IL-1β, and IL-6 [43]. This may explain the anti-inflammatory effect observed with ebselen, an organoselenium compound. In chronic wounds (such as diabetic PUs), wound healing can stall in the inflammation stage and not proceed to re-epithelization of damaged tissue [15, 44]. Individuals that are obese or diabetic are more susceptible to chronic, non-healing wounds due to being in an extended inflammatory state [15, 44]. Given selenium-based compounds like ebselen have been shown to inhibit expression of pro-inflammatory cytokines, this may promote healing of infected pressure ulcers (or lead to remission), an advantage not present with currently approved antibiotics. In addition to its potent antibacterial activity against MRSA, ebselen has also been shown to be safe when exposed to human keratinocytes (HaCaT) *in vitro* (IC_50_ = 58.78 μg/mL) [26]. Taking the potent antibacterial, antibiofilm, anti-inflammatory activities and safety profile into consideration, we hypothesized that ebselen would be a promising new topical agent to treat mild-to-moderate pressure ulcers infected with MRSA in high-risk populations, namely those that are obese and/or diabetic.

Due to concern about bacterial resistance emerging rapidly to new antibacterial agents, we first investigated the likelihood of *S*. *aureus* to develop resistance to ebselen. Previously Gustafsson, *et al*. conducted a single-step resistance selection for ebselen against *S*. *aureus* ATCC 29213 and were unable to isolate resistant mutants to ebselen (at 8 × MIC) [45]. Furthermore, they evaluated if resistance to ebselen would emerge after bacteria were repeatedly exposed to the compound via a multi-step resistance selection experiment. No shift in MIC was observed [45]. However, it should be noted that Gustafsson, *et al*. used a single strain of *S*. *aureus* susceptible to β-lactams, and the study was only conducted for five passages. Current guidelines for the treatment of mild-to-moderate PUs infected with bacteria recommend patients receive one-to-two weeks of antibiotics [9, 15]. With this in mind, we conducted a multi-step resistance selection experiment where ebselen was serially passaged with two different strains of *S*. *aureus* (including one MRSA strain) for two weeks. Neither strain of *S*. *aureus* was able to develop resistance to ebselen. The lack of resistance formation observed for *S*. *aureus* to ebselen aligns with a recent study that determined another important Gram-positive pathogen, vancomycin-resistant *Enterococcus faecium*, was unable to develop resistance to ebselen after 14 consecutive passages [21].

Ebselen was subsequently evaluated for the ability to reduce the burden of MRSA in infected PUs in obese and diabetic mice. Previous reports have demonstrated that topical ebselen significantly reduced the burden of MRSA (≥ 1.71-log_10_ reduction) in cutaneous abscesses in mice and rats after only five days of topical treatment (two doses daily) [23, 26]. Based upon this information, we decided to treat MRSA-infected PUs formed in obese mice topically with ebselen twice daily for five days. Though ebselen did produce a statistically significant reduction in the burden of MRSA in PUs in obese mice, it was less effective than topical mupirocin at the same concentration. When a similar experiment was conducted in diabetic mice with MRSA-infected pressure ulcers, ebselen was unable to produce a statistically significant reduction in bacterial burden relative to the vehicle alone. These results were similarly supported by histopathology treatment demonstrating ebselen and mupirocin treatment decreased the bacterial burden deep within the skin and surrounding tissues, compared to untreated controls. In both treatment groups, necrosuppurative inflammation remained pronounced, acute, and marked, with a mild decrease in the depth and severity of lesions compared to untreated controls. A small amount of early wound regeneration was present in both treated groups, with few infiltrating macrophages, lymphocytes, and plasma cells or mild peripheral re-epithelization of ulcerated margins. Resolution of dermal necrosis was patchy in mupirocin-treated animals (Fig 2D) and was more consistent and widespread in ebselen-treated animals compared to untreated controls. In both treatment groups, mild epithelial regeneration was present, indicting early wound healing compared to untreated controls. Both treatment groups had animals with mild to moderate mineralization, which extended more deeply in the ebselen-treated groups (S1 Table).

The reduced efficacy observed for ebselen in treating MRSA-infected PUs in obese and diabetic mice was surprising given previous reports of the effectiveness of topical ebselen in reducing the burden of MRSA in simple abscesses in rodents [23, 26]. We suspected that the lack of efficacy may be due to the impact of pH in the wound bed. Healthy skin typically is acidic and the pH on the skin surface ranges from 4.0 to 6.0 [31]. However, acute skin wounds tend to exhibit a more neutral pH (~7.4), similar to the pH used in media for standard antibacterial susceptibility assays [31]. In chronic wounds, such as PUs, the pH of the wound bed becomes more basic (pH ranges from 7.15 to 8.93) [31]. This change in pH could potentially impact the antibacterial activity of topical drugs. For example, mupirocin has been found to exhibit more potent antibacterial activity (lower MIC) against *S*. *aureus* under acidic conditions [32, 33]. To assess if ebselen’s antibacterial activity would be negatively impacted by a change in pH, the standard broth microdilution assay was used with acidic, neutral, and basic media. Interestingly, the MIC for ebselen was not impacted by adjusting the pH to acidic (pH 6.0) or basic (pH 8.0) conditions.

Next, we evaluated the impact of bacterial inoculum size on the antibacterial activity of ebselen. The inoculum effect is a phenomenon observed for certain antibiotics that as the bacterial count is increased, usually ≥100-fold compared to standard broth microdilution assay conditions (where 10^5^ CFU/mL bacteria are used), the efficacy of the antibiotic is decreased (e.g. a higher concentration of drug would be needed to achieve the same effect against a higher inoculum size) [46]. This effect has been observed for numerous antibiotic classes including for β-lactams, glycopeptides, aminoglycosides, and quinolones [46]. This can impact the size of dose that needs to be administered to successfully eradicate an infection. Previously, Ngo, *et al*. evaluated the effect of increasing *S*. *aureus* inoculum size from 10^5^ to 10^6^ CFU/mL on ebselen’s antibacterial activity and found no change in the MIC values *in vitro* [41]. However, as noted above, typically the inoculum effect is observed when the bacterial count is increased ≥100-fold compared to conditions used in the standard broth microdilution assay. This is important given Konig, *et al*., determined that bacteria typically exceeded 10^8^ CFU/mL in the pus of soft-tissue infections [47]. Thus, we investigated the effect of increasing the inoculum size of *S*. *aureus* count on the antibacterial activity of ebselen. When the bacterial count was increased to 10^7^ CFU/mL (irrespective of the change in pH), a significant loss in antibacterial activity was observed for ebselen (MIC increased 32- to 64-fold against all three strains of *S*. *aureus* tested). Given a high inoculum of MRSA USA300 was used in the mice study, this may partially explain the weak activity observed for ebselen in the MRSA-infected PU mice studies. Thus, a higher concentration of ebselen may be needed to achieve a similar effect as observed with topical mupirocin. Additionally, it is important to note that different strains of mice were used in our study (TALLYHO/JngJ) compared to the previous study (BALB/c) evaluating ebselen treatment of cutaneous abscesses infected with MRSA [26]. Furthermore, mice used in this study were obese and/or diabetic which is important given that the pathology and biomechanical properties of skin in nondiabetic and diabetic mice is different [48]. This change in pathology may have impacted the activity of ebselen *in vivo*. The difference in activity observed between ebselen in the treatment of infected PUs for obese (female) and diabetic (male) mice could be due to a difference in sex, due to diabetic mice receiving one day less of treatment (due to concern about morbidity observed in one mouse in the ebselen treatment group), or the small number of mice used in each experiment. As obesity is a major risk factor associated with type 2 diabetes for both men and women, the difference in sex is an important variable to consider given that more women are categorized as being obese worldwide [49]. However, type 2 diabetes impacts men more commonly at a younger age, and subsequently adult men are at higher risk for developing diabetic foot ulcers that result in amputation of a lower limb [50–52]. A future study investigating the effect of ebselen in the treatment of infected diabetic pressure ulcers in male versus female mice would be of interest. As such a study is not feasible with TALLYHO/JngJ mice, another strain of mice (such as db/db mice) would need to be used. Altogether, the results suggest ebselen, at the dose and duration studied in mice, is not a better candidate than currently available antibiotics to treat MRSA-infected pressure ulcers. A higher dose or longer course of treatment with ebselen could potentially address the lack of efficacy observed *in vivo*, though a future study would need to be conducted to evaluate this further.

## Conclusions

Bacterial infected-pressure ulcers are a source of morbidity in individuals with limited mobility including individuals that are obese and diabetic. Ebselen was evaluated as a novel topical antibacterial agent to treat MRSA-infected PUs in mice. No detectable formation of resistance to ebselen by MRSA was observed *in vitro* in a multi-step resistance selection assay. Additionally, ebselen exhibited a slightly longer postantibiotic effect than mupirocin against MRSA. In obese and diabetic mice with MRSA-infected PUs, ebselen was found to be less effective than mupirocin in reducing the burden of bacteria. The lack of efficacy observed may be due to the high bacterial burden present within the PUs suggesting a higher dose or longer course of treatment with ebselen may be needed. Overall, the results from this study indicate topical ebselen may not be a favorable candidate to pursue for short-term treatment of infected chronic wounds such as pressure ulcers infected with MRSA.

## Materials and methods

### Antibiotics and reagents

Clinical isolates of *S*. *aureus* were obtained from the American Type Culture Collection (ATCC, Manassas, VA, USA) or the Biodefense and Emerging Infections Research Resources Repository (BEI Resources, Manassas, VA, USA). Ebselen, linezolid, and mupirocin were purchased from commercial vendors and dissolved in DMSO to prepare 10 mg/mL (or 1 mg/mL for ebselen) stock solutions. Cation-adjusted Mueller Hinton broth (CA-MHB), Tryptic soy agar (TSA), Tryptic soy broth (TSB), mannitol salt agar, phosphate-buffered saline (PBS), hydrochloric acid (HCl), sodium hydroxide (NaOH), petroleum jelly, magnets, Betadine®, buprenorphine, Tegaderm, Uro-bond IV, and 96-well plates were all purchased from commercial vendors.

### Multi-step resistance selection experiment for *S*. *aureus* against ebselen

To investigate the ability of *S*. *aureus* to develop resistance to ebselen after multiple exposures, a multi-step resistance experiment was conducted, as described previously [30, 53, 54]. Plates containing bacteria and drugs were incubated at 37°C for at least 20 hours before the MIC was determined by visual inspection for growth. Bacteria were passaged with ebselen or mupirocin for 14 days and resistance was characterized as a >4-fold increase in MIC relative to the initial MIC (passage 0) [28].

### Postantibiotic effect (PAE) of ebselen against *S*. *aureus*

The PAE for ebselen was determined as described in a previous study [30]. The PAE was calculated via the equation: *T* – *C*, where *T* represents the time required for *S*. *aureus* exposed to either ebselen or mupirocin to increase by one log_10_ (after the test agent was washed out) and *C* represents the time for the bacterial inoculum in the untreated control to increase by one log_10_.

### MRSA-infected PU obese mouse model

All mice studies were approved by the Purdue Animal Care and Use Committee (Protocol #: 170400567 and Protocol #: 1704001564) and conducted in accordance with the National Institutes of Health Guide for the Care and Use of Laboratory Animals. Mice were checked every four hours for signs of morbidity (hypothermia, hunched posture, decreased activity, and inability to eat or drink) during the duration of both mice studies and all efforts were made to minimize suffering. For the obese mouse model study, twelve-week-old, female, TALLYHO/JngJ mice (Jackson Laboratory, Bar Harbor, ME, USA) (JAX stock #005314), weighing 27–35 grams, were housed in ventilated cages with access to water and food *ad libitum*. To induce the formation of MRSA-infected PUs in mice, a previously published method was used with the following changes [30]. To minimize pain during the application of rare-earth magnets, buprenorphine was administered to mice twice daily. In pilot studies, we determined that pressure ulcers in mice infected with 1 × 10^7^ to 2 × 10^7^ CFU MRSA USA300 exhibited similar morphological features to infected pressure ulcers observed in human patients. Additionally, at this particular infectious dose, mice were unable to clear the infection on their own without antibiotic treatment. Thus, in both the obese and diabetic mice studies, we aimed to infect the pressure ulcers with MRSA USA300 at an infectious dose that ranged between 1 × 10^7^ to 2 × 10^7^ CFU. After the formation of PUs on obese mice using rare-earth magnets, ulcers were infected with 1.28×10^7^ CFU MRSA NRS384 (USA300) and covered with Tegaderm. This strain was selected as it is a leading source of MRSA skin-and-soft tissue infections, is capable of forming biofilms, and secretes multiple toxins including PVL [55–57]. Two days post-infection, before starting treatment, mice were randomly divided into groups of five (n = 5). One group of mice was treated orally with linezolid (10 mg/kg) twice daily for five days while remaining groups were treated twice daily for five days with a topical preparation consisting of 2% ebselen, 2% mupirocin, or the vehicle alone (petroleum jelly). Mupirocin, when used topically to treat bacterial skin infections, is prepared as a 2% w/w ointment [58, 59]. In order to compare the effectiveness of topical ebselen relative to mupirocin, we chose to test both molecules at the same concentration (2% w/w). Previously, our research group has found that a 2% w/w dose of topical ebselen applied twice daily is effective at reducing the burden of MRSA in an uncomplicated skin abscess mouse model (using Balb/c mice) [26]. Thus, for the MRSA pressure ulcer mice studies, we chose to use 2% w/w topical ebselen as the treatment dose. Mice were humanely euthanized 12 hours after the last dose via CO_2_ asphyxiation. The ulcerated tissue was aseptically harvested and homogenized in sterile PBS using an Omni Tissue Homogenizer (TH115, Omni International, Kennesaw, GA, USA). The homogenate was serially diluted in PBS and aliquots (4 μL) of each dilution were plated on mannitol salt agar plates (to select for *S*. *aureus* colonies). Plates were incubated for at least 20 hours at 37°C before bacterial colonies were enumerated. Data are presented as log_10_ (MRSA CFU) from each PU.

### MRSA-infected PU diabetic mouse model

Twelve-week-old, male, TALLYHO/JngJ mice (Jackson Laboratory, Bar Harbor, ME, USA), weighing on average 34.9 grams, were used for this experiment. Mice were fed a high fat diet to induce the diabetes phenotype. Female TALLYHO/JngJ mice do not develop characteristic features of diabetes (e.g. hyperglycemia), as per correspondence with representatives at The Jackson Laboratory, which is why male mice were used. Two days prior to application of magnets, the blood glucose was measured for all mice using a standard glucose home monitoring kit (concentrations >125 mg/dL for mice in a fasting state were categorized as diabetic, per the manufacturer’s guidelines). Pressure ulcers were induced and infected with 1.8 × 10^7^ CFU MRSA USA300, as described above. Infected PUs were treated topically twice daily for four days with the vehicle alone, 2% ebselen, or 2% mupirocin. Mice were checked every four hours for signs of morbidity (hypothermia, hunched posture, decreased activity, and inability to eat or drink) during the duration of the study and all efforts were made to minimize suffering. The decision to only treat for four days was made due to signs of morbidity (hypothermia and decreased activity) observed in one mouse in the ebselen treatment group. Twelve hours after the last dose was administered, mice were euthanized as described above and ulcerated tissues were harvested aseptically. The ulcerated tissue (right wound) was aseptically harvested, homogenized in sterile PBS, serially diluted in PBS, and plated on mannitol salt agar plates. Plates were incubated for at least 20 hours at 37°C before bacterial colonies were enumerated. Data are presented as log_10_ (MRSA CFU) from each PU.

### Histopathological evaluation of infected pressure ulcers in diabetic mice

Pressure ulcers (left wound) from infected mice in all three groups of diabetic mice (vehicle, ebselen, and mupirocin) were aseptically harvested after mice were euthanized and evaluated histologically. Sections of affected skin were removed en bloc and placed in room temperature 10% neutral-buffered formalin for 24 hours. Tissues were processed over 10 hours using a Sakura Tissue-Tek VIP6 tissue processor. Tissues were processed sequentially in 70%, 80%, 95%, and 100% ethanol, followed by xylene and paraffin, and were embedded in Surgipath Paraplast Plus (Leica Biosystems, Wetzlar, Germany). Tissue sections, 4-mm thickness, were placed on charged slides, stained with hematoxylin and eosin, and cover- slipped using a Leica ST5010-CV5030 integrated workstation.

### Impact of pH and inoculum size on the antibacterial activity of ebselen

The broth microdilution assay was used to determine the minimum inhibitory concentration (MIC) of ebselen and mupirocin against three different clinical isolates of *S*. *aureus* [60]. To evaluate the effect of pH on antibacterial activity, either 1 M HCl or 1 M NaOH was added to CA-MHB until a pH of 6.00 ± 0.1, 8.00 ± 0.1, or 9.00 ± 0.1 was reached. To determine the inoculum effect on the antibacterial activity of ebselen and mupirocin, a preparation of *S*. *aureus* equivalent to McFarland 0.5, in sterile PBS, was subsequently diluted 1:3 (to reach 10^7^ CFU/mL) in sterile CA-MHB (pH 7.4). Ebselen or mupirocin was added to wells (in triplicate) to a 96-well plate and serially diluted. Bacteria were subsequently added to wells. Plates were incubated at 37°C for 18–20 hours before the MIC was recorded by visual inspection of growth. The experiment was conducted twice to confirm the results.

### Statistical analyses

Data from the pressure ulcer mice studies were analyzed via a one-way ANOVA with post-hoc Dunnet’s test for multiple comparisons (*P* < 0.05) using GraphPad Prism8 (La Jolla, CA). The Kolmogorov-Smirnov test (*P* < 0.05) was utilized to confirm the data exhibited a normal distribution.

## Supporting information

S1 Table Grading scheme for severity of lesions in each animal in the negative control, mupirocin-treated, or ebselen-treated groups. (DOCX)
