# Supplementary material for: Evaluation of ebselen in resolving a methicillin-resistant Staphylococcus aureus infection of pressure ulcers in obese and diabetic mice
Source: PLoS One. 2021 Feb 22;16(2):e0247508. doi: 10.1371/journal.pone.0247508 (PMC7899319; doi:10.1371/journal.pone.0247508)
Supplement: S1 Table — (DOCX) [file pone.0247508.s001.docx]

**S1 Table.** **Grading scheme for severity of lesions in each animal in the negative control, mupirocin-treated, or ebselen-treated groups.**

|  | **Bacterial burden^1^** | **Inflammation^2^** | **Early wound healing^3^** | **Necrosis^4^** | **Mineralization^5^** |
| --- | --- | --- | --- | --- | --- |
| Negative controls | | | | | |
| V1 | 4+ | 5 | 0 | 5* | 0 |
| V2 | 5++ | 5 | 0 | 5* | 0 |
| V3 | 2+ | 5 | 0 | 5 | 0 |
| V4 | 2+ | 5 | 0 | 5* | 0 |
| V5 | 3++ | 5 | 0 | 5 | 0 |
| V Group | 3.2+ | 5 | 0 | 5* | 0 |
| Mupirocin-treated | | | | | |
| M1 | 2 | 4 | 2 | 5 | 1 |
| M2 | 2 | 4 | 2 | 5 | 0 |
| M3 | 2 | 4 | 0 | 5 | 0 |
| M4 | 2 | 5 | 1 | 5 | 0 |
| M5 | 2 | 5 | 0 | 5 | 0 |
| M Group | 2 | 4.4 | 1 | 5 | 0 |
| Ebselen-treated | | | | | |
| E1 | 2 | 5 | 0 | 3 | 0 |
| E2 | 2 | 5 | 0 | 3 | 0 |
| E3 | 2 | 4 | 0 | 4 | 2 |
| E4 | 2 | 3 | 2 | 3 | 0 |
| E5 | 2 | 5 | 2 | 4 | 0 |
| E Group | 2 | 4.4 | 1 | 3.4 | 0 |

Grading scheme for bacterial burden, inflammation, wound healing, necrosis, and mineralization. In all experimental groups examined, the epidermis and dermis were ulcerated. V1-5 represent vehicle-treated negative controls, M1-5 represent mupirocin-treated animals, and E1-5 are ebselen-treated animals (n=5): Group represents mean score for treatment group).

^1^Bacterial burden: 0 = none, 1 = confined to superficial ulcerated regions, 2 = extends into dermis, folliculocentric, 3 = extends into subcutis, 4 = extends deep into the subcutis, 5 = full thickness panniculitis/myositis in deep muscle + = numerous bacterial colonies, ++ = innumerable.

^2^Inflammation (acute/neutrophilic): 0 = none, 1 = mild, superficial dermis, 2 = moderate, superficial dermis/folliculocentric, 3 = moderate deep subcutis, 4 = marked, deep subcutis/ panniculus, 5 = severe, deep subcutis/panniculus.

^3^Early wound healing: 0 = no wound healing, 1 = peripheral wound contraction with re-epithelialization, 2 = re-epithelialization at wound margins, 3 = moderate numbers of lymphocytes, plasma cells, macrophages, 4 = mild numbers of lymphocytes and plasma cells, 5 = resolution of inflammation.

^4^Necrosis: 0 = none, 1 = confined to epidermis, 2 = within dermis, epidermis intact, 3 = marked within subcutis dermis, collagen intact, 4 = marked extending into subcutis, 5 = extends full thickness into deep subcutis/skeletal muscle, *pronounced superficial crust with bacteria and neutrophilic debris.

^5^Mineralization: 0 = none, 1 = mild, epidermis/superficial dermis, 2 = moderate, epidermis/superficial dermis, 3 = mild, subcutis, 4 = moderate, deep skeletal muscle, 5 = marked, subcutis.
